# 2-Bromo-1,6,6-trimethyl-6,7,8,9-tetra­hydro­phenanthro[1,2-*b*]furan-10,11-dione

**DOI:** 10.1107/S1600536813011483

**Published:** 2013-05-04

**Authors:** Cui-Ping Fan, Wei-Ping Yin, Xin-Xiang Cao, Jing-Cai Yao

**Affiliations:** a Chemical Engineering & Pharmaceutics College, Henan University of Science and Technology, Luoyang, Henan 471003, People’s Republic of China; b College of Chemistry and Chemical Engineering, Luoyang Normal University, Luoyang, Henan 471022, People’s Republic of China

## Abstract

In the title compound, C_19_H_17_BrO_3_, the ring skeleton is located on a crystallographic mirror plane; two C atoms of the cyclo­hexene ring are disordered over the two locations to satisfy the preferred ring conformation. In the crystal, C—H⋯O hydrogen bonds link the mol­ecules into chains along the *a* axis. π–π stacking inter­actions between benzo­quinone rings, with a centroid–centroid distance of 3.7225 (4) Å, are also observed, which connect the chains into a two-dimensional networkparallel to the *ab* plane.

## Related literature

The title compound is a derivative of Tanshinone IIA, the major active component isolated from the Chinese herbal medicine danshen, which is used in the treatment of coronary heart disease (Chang *et al.*, 1991 ▶; Wang *et al.*, 2005 ▶), myocard­ial infarction and angina pectoris (Xue *et al.*, 1999 ▶) and has anti­tumour activity (Ryu *et al.*, 1997 ▶). For the structure of 1,6,6-trimethyl-6,7,8,9-tetra­hydro­phenanthro[1,2-*b*]furan-10,11-dione, see: Liu & Gao (2007 ▶).
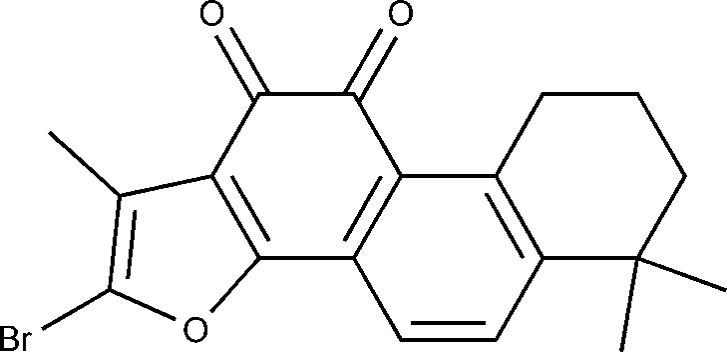


## Experimental

### 

#### Crystal data

C_19_H_17_BrO_3_
*M* *_r_* = 373.24
Monoclinic, 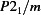

*a* = 9.6063 (12) Å
*b* = 7.0457 (9) Å
*c* = 11.9688 (15) Å
β = 96.723 (1)°
*V* = 804.52 (18) Å^3^
*Z* = 2
Mo *K*α radiation
μ = 2.57 mm^−1^
*T* = 296 K
0.48 × 0.15 × 0.12 mm

#### Data collection

Bruker SMART CCD area-detector diffractometer
Absorption correction: multi-scan (*SADABS*; Bruker, 2005 ▶) *T*_min_ = 0.372, *T*_max_ = 0.748
6178 measured reflections
1634 independent reflections
1177 reflections with *I* > 2σ(*I*)
*R*_int_ = 0.046

#### Refinement

*R*[*F* ^2^ > 2σ(*F* ^2^)] = 0.033
*wR*(*F* ^2^) = 0.087
*S* = 1.02
1634 reflections
144 parameters
H-atom parameters constrained
Δρ_max_ = 0.22 e Å^−3^
Δρ_min_ = −0.41 e Å^−3^

### 

Data collection: *SMART* (Bruker, 2005 ▶); cell refinement: *SAINT* (Bruker, 2005 ▶); data reduction: *SAINT*; program(s) used to solve structure: *SHELXS97* (Sheldrick, 2008 ▶); program(s) used to refine structure: *SHELXL97* (Sheldrick, 2008 ▶); molecular graphics: *SHELXTL* (Sheldrick, 2008 ▶); software used to prepare material for publication: *SHELXTL*.

## Supplementary Material

Crystal structure: contains datablock(s) global, I. DOI: 10.1107/S1600536813011483/kp2449sup1.cif

Structure factors: contains datablock(s) I. DOI: 10.1107/S1600536813011483/kp2449Isup2.hkl

Additional supplementary materials:  crystallographic information; 3D view; checkCIF report

## Figures and Tables

**Table 1:** Hydrogen-bond geometry (Å, °)

*D*—H⋯*A*	*D*—H	H⋯*A*	*D*⋯*A*	*D*—H⋯*A*
C6—H6⋯O2^i^	0.93	2.39	3.322 (4)	177

Symmetry code: (i) 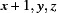.

**Fig. 1.**
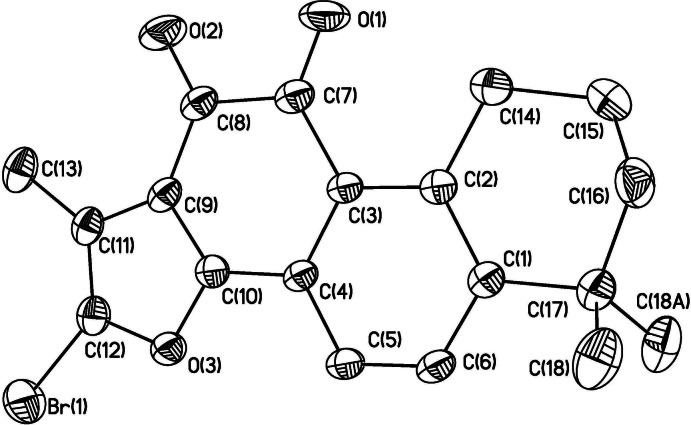
The molecular structure of (I), with atom labels and 50% probability displacement ellipsoids for non-H atoms. C15, C16 atoms are in disorder over two locations but one for each atom is shown. Symmetry code used for (C18a): x,-y+3/2,z.

## supplementary crystallographic information

### Comment

The Chinese herbal medicine, danshen, comes from the dried root of Salvia miltiorrhiza Bunge and Salvia przewalskii Maxim(Labiatae). Tanshinone IIA is the major active component isolated from danshen, which has unique curative effect in treating coronary heart disease (Chang *et al.*, 1991; Wang *et al.*, 2005), antitumour (Ryu *et al.*, 1997), myocardial infarction and angina pectoris (Xue *et al.*, 1999). The title compound, C_19_H_17_BrO_3_, is derivative obtained by modification of Tanshinone IIA and may be used for obtaining molecules with higher bioactivity and better solubility.

The crystal structure of (I) contains three six-membered rings forming a phenanthrene dione system with a five-membered methylfuran ring fused to the dione ring (Fig. 1). The bond distance of C7-C8 agree with the corresponding distance of 1.564 (3) Å reported for 1,6,6-trimethyl-6,7,8,9-tetrahydrophenanthro[1,2-b]furan-10,11- -dione (Liu *et al.*, 2007), indicating the nonconjugation system of the C3, C4, C7-C10 ring. The bond distance of Br1-C12 is 1.852 (3) Å. The ring skeleton is located at the crystallographic mirror plane except the atoms C15 and C16 which are disordered over two locations. Thus, the terminal six-membered ring is in a twist form with torsion angles C1-C2-C14-C15 = 19.8 (2)°, C1-C2-C16-C17 = 13.5 (2)°, the deviations of C15, C16, C18, C18A from the least square plane are 0.4867Å, -0.3383Å, -1.2396Å and 1.2396Å, respectively. Intermolecular C—H···O hydrogen bonds of d(O2···C6) = 3.322 (4) Å link the title molecules into a one-dimensional chain along the a axis (Table 1). The pi-pi stacking interactions between benzoquinone rings with the centroid-centroid distance of 3.7225 (4) Å [symmetry operations involved: 1-x,-1/2+y,-z;1-x,1/2+y-z;1-x,-1-y,-z;1-x,2-y,-z] are observed in the crystal structure.

### Experimental

Tanshinone IIA (0.3 mmol) was added to dry dichloromethane(15 mL)in a three neck flask. The mixture was stirred and was heated to reflux temperature. And then, N-bromosuccinimide(0.36mmol) and benzoyl peroxide(0.03mmol) were dropped into the flask. After reflux reaction for 9h and left stirring about 17h at room temperature, the solvent in flask was evaporated. The residue was purified by column chromatography on silica gel with ethyl acetate/petroleum ether to afford the title compound solid (109 mg, yield 97.08%). The crimson crystals of the title compound for structure determination were obtained from recrystallization of the product from ethyl acetate at room temperature.

### Refinement

H1W and H2W were located by a difference map and refined isotropically. All of the remaining H atoms were positioned geometrically and treated as riding, with C—H bonding lengths constrained to 0.93 Å (aromatic CH) or 0.97 Å (methylene CH_2_), and with *U*_iso_(H) = 1.2*U*_eq_(C) or 1.5*U*_eq_ (methylene C).The carbon atoms of C15 and C16 located from the terminal cyclohexene ring and five hydrogen atoms H13a, H13b, H13c, H14a, H14b were observed disordered at two close positions with the half occupancy, respectively.

### Crystal data

**Table:**

C_19_H_17_BrO_3_	*F*(000) = 380
*M**_r_* = 373.24	*D*_x_ = 1.541 Mg m^−^^3^
Monoclinic, *P*2_1_/*m*	Mo *K*α radiation, λ = 0.71073 Å
Hall symbol: -P 2yb	Cell parameters from 2150 reflections
*a* = 9.6063 (12) Å	θ = 2.6–23.0°
*b* = 7.0457 (9) Å	µ = 2.57 mm^−^^1^
*c* = 11.9688 (15) Å	*T* = 296 K
β = 96.723 (1)°	Block, brown
*V* = 804.52 (18) Å^3^	0.48 × 0.15 × 0.12 mm
*Z* = 2	

### Data collection

**Table:**

Bruker SMART CCD area-detector diffractometer	1634 independent reflections
Radiation source: fine-focus sealed tube	1177 reflections with *I* > 2σ(*I*)
Graphite monochromator	*R*_int_ = 0.046
phi and ω scans	θ_max_ = 25.5°, θ_min_ = 2.6°
Absorption correction: multi-scan (*SADABS*; Bruker, 2005)	*h* = −11→11
*T*_min_ = 0.372, *T*_max_ = 0.748	*k* = −8→8
6178 measured reflections	*l* = −14→14

### Refinement

**Table:**

Refinement on *F*^2^	Primary atom site location: structure-invariant direct methods
Least-squares matrix: full	Secondary atom site location: difference Fourier map
*R*[*F*^2^ > 2σ(*F*^2^)] = 0.033	Hydrogen site location: inferred from neighbouring sites
*wR*(*F*^2^) = 0.087	H-atom parameters constrained
*S* = 1.02	*w* = 1/[σ^2^(*F*_o_^2^) + (0.0469*P*)^2^] where *P* = (*F*_o_^2^ + 2*F*_c_^2^)/3
1634 reflections	(Δ/σ)_max_ < 0.001
144 parameters	Δρ_max_ = 0.22 e Å^−^^3^
0 restraints	Δρ_min_ = −0.41 e Å^−^^3^

### Special details

**Table:**

Geometry. All esds (except the esd in the dihedral angle between two l.s. planes) are estimated using the full covariance matrix. The cell esds are taken into account individually in the estimation of esds in distances, angles and torsion angles; correlations between esds in cell parameters are only used when they are defined by crystal symmetry. An approximate (isotropic) treatment of cell esds is used for estimating esds involving l.s. planes.
Refinement. Refinement of F^2^ against ALL reflections. The weighted R-factor wR and goodness of fit S are based on F^2^, conventional R-factors R are based on F, with F set to zero for negative F^2^. The threshold expression of F^2^ > 2sigma(F^2^) is used only for calculating R-factors(gt) etc. and is not relevant to the choice of reflections for refinement. R-factors based on F^2^ are statistically about twice as large as those based on F, and R- factors based on ALL data will be even larger.

### Fractional atomic coordinates and isotropic or equivalent isotropic displacement parameters (Å^2^)

**Table:**

	*x*	*y*	*z*	*U*_iso_*/*U*_eq_	Occ. (<1)
Br1	0.50666 (4)	0.7500	−0.40805 (3)	0.0774 (2)	
C1	0.9138 (3)	0.7500	0.2002 (2)	0.0392 (7)	
C2	0.7711 (3)	0.7500	0.2173 (2)	0.0365 (7)	
C3	0.6680 (3)	0.7500	0.1229 (2)	0.0354 (7)	
C4	0.7082 (3)	0.7500	0.0134 (2)	0.0371 (7)	
C5	0.8494 (3)	0.7500	−0.0014 (3)	0.0462 (8)	
H5	0.8766	0.7500	−0.0734	0.055*	
C6	0.9483 (3)	0.7500	0.0909 (3)	0.0471 (8)	
H6	1.0425	0.7500	0.0798	0.057*	
C7	0.5146 (3)	0.7500	0.1357 (3)	0.0456 (8)	
C8	0.4040 (3)	0.7500	0.0286 (3)	0.0440 (8)	
C9	0.4585 (3)	0.7500	−0.0783 (3)	0.0405 (8)	
C10	0.5998 (3)	0.7500	−0.0809 (3)	0.0387 (7)	
C11	0.3933 (3)	0.7500	−0.1926 (3)	0.0435 (8)	
C12	0.5002 (4)	0.7500	−0.2540 (3)	0.0490 (8)	
C13	0.2392 (4)	0.7500	−0.2319 (3)	0.0590 (10)	
H13A	0.2017	0.8744	−0.2222	0.088*	0.50
H13B	0.1930	0.6601	−0.1886	0.088*	0.50
H13C	0.2244	0.7155	−0.3100	0.088*	0.50
C14	0.7287 (4)	0.7500	0.3362 (3)	0.0513 (9)	
H14A	0.7133	0.6252	0.3353	0.062*	0.50
H14B	0.6436	0.8237	0.3530	0.062*	0.50
C15	0.8496 (5)	0.8191 (7)	0.4235 (4)	0.0587 (15)	0.50
H15B	0.8751	0.9509	0.4144	0.070*	0.50
H15A	0.8219	0.8036	0.4983	0.070*	0.50
C16	0.9772 (5)	0.7020 (8)	0.4095 (3)	0.058 (2)	0.50
H16A	0.9494	0.5696	0.4079	0.070*	0.50
H16B	1.0497	0.7207	0.4721	0.070*	0.50
C17	1.0334 (3)	0.7500	0.2971 (3)	0.0495 (8)	
C18	1.1253 (3)	0.5741 (4)	0.2885 (2)	0.0726 (8)	
H18A	1.1998	0.5740	0.3494	0.109*	
H18B	1.1641	0.5760	0.2182	0.109*	
H18C	1.0694	0.4619	0.2925	0.109*	
O1	0.4673 (3)	0.7500	0.2239 (2)	0.0881 (10)	
O2	0.2812 (3)	0.7500	0.0408 (2)	0.0763 (8)	
O3	0.6299 (2)	0.7500	−0.18897 (17)	0.0468 (6)	

### Atomic displacement parameters (Å^2^)

**Table:**

	*U*^11^	*U*^22^	*U*^33^	*U*^12^	*U*^13^	*U*^23^
Br1	0.0776 (4)	0.1056 (4)	0.0470 (3)	0.000	−0.0020 (2)	0.000
C1	0.0356 (18)	0.0377 (18)	0.0443 (18)	0.000	0.0051 (14)	0.000
C2	0.0394 (18)	0.0273 (16)	0.0436 (18)	0.000	0.0093 (14)	0.000
C3	0.0328 (17)	0.0327 (16)	0.0422 (17)	0.000	0.0107 (14)	0.000
C4	0.0293 (17)	0.0391 (17)	0.0435 (17)	0.000	0.0072 (14)	0.000
C5	0.0348 (19)	0.063 (2)	0.0420 (18)	0.000	0.0106 (15)	0.000
C6	0.0283 (17)	0.064 (2)	0.050 (2)	0.000	0.0080 (14)	0.000
C7	0.0374 (19)	0.0464 (19)	0.0548 (19)	0.000	0.0126 (16)	0.000
C8	0.0323 (19)	0.0402 (18)	0.060 (2)	0.000	0.0093 (16)	0.000
C9	0.0304 (18)	0.0346 (17)	0.056 (2)	0.000	0.0041 (15)	0.000
C10	0.0386 (19)	0.0378 (17)	0.0401 (18)	0.000	0.0062 (14)	0.000
C11	0.0397 (19)	0.0343 (18)	0.055 (2)	0.000	−0.0023 (16)	0.000
C12	0.046 (2)	0.051 (2)	0.048 (2)	0.000	−0.0043 (17)	0.000
C13	0.045 (2)	0.047 (2)	0.080 (3)	0.000	−0.0106 (19)	0.000
C14	0.048 (2)	0.061 (2)	0.0461 (19)	0.000	0.0119 (16)	0.000
C15	0.069 (3)	0.066 (4)	0.042 (3)	0.001 (2)	0.012 (2)	−0.005 (2)
C16	0.060 (3)	0.069 (8)	0.042 (2)	−0.004 (3)	−0.008 (2)	0.006 (2)
C17	0.0395 (19)	0.059 (2)	0.049 (2)	0.000	0.0007 (15)	0.000
C18	0.0569 (17)	0.0664 (19)	0.089 (2)	0.0088 (14)	−0.0157 (14)	0.0102 (15)
O1	0.0423 (15)	0.174 (3)	0.0517 (16)	0.000	0.0206 (13)	0.000
O2	0.0311 (15)	0.124 (2)	0.0759 (18)	0.000	0.0144 (13)	0.000
O3	0.0378 (13)	0.0616 (15)	0.0409 (13)	0.000	0.0045 (10)	0.000

### Geometric parameters (Å, º)

**Table:**

Br1—C12	1.852 (3)	C13—H13A	0.9600
C1—C6	1.386 (4)	C13—H13B	0.9600
C1—C2	1.410 (4)	C13—H13C	0.9600
C1—C17	1.534 (4)	C14—C15^i^	1.547 (5)
C2—C3	1.412 (4)	C14—C15	1.547 (5)
C2—C14	1.526 (4)	C14—H14A	0.8918
C3—C4	1.409 (4)	C14—H14B	1.0084
C3—C7	1.499 (4)	C15—C15^i^	0.973 (9)
C4—C5	1.388 (4)	C15—C16^i^	1.265 (6)
C4—C10	1.444 (4)	C15—C16	1.503 (6)
C5—C6	1.371 (4)	C15—H15B	0.9700
C5—H5	0.9300	C15—H15A	0.9700
C6—H6	0.9300	C16—C16^i^	0.677 (12)
C7—O1	1.197 (4)	C16—C15^i^	1.265 (6)
C7—C8	1.566 (5)	C16—C17	1.545 (5)
C8—O2	1.205 (4)	C16—H16A	0.9700
C8—C9	1.439 (4)	C16—H16B	0.9700
C9—C10	1.361 (4)	C17—C18^i^	1.532 (3)
C9—C11	1.436 (4)	C17—C18	1.532 (3)
C10—O3	1.358 (3)	C17—C16^i^	1.545 (5)
C11—C12	1.331 (5)	C18—H18A	0.9600
C11—C13	1.499 (4)	C18—H18B	0.9600
C12—O3	1.389 (4)	C18—H18C	0.9600
			
C6—C1—C2	118.7 (3)	C15—C14—H14B	105.4
C6—C1—C17	118.2 (3)	H14A—C14—H14B	111.9
C2—C1—C17	123.1 (3)	C15^i^—C15—C16^i^	83.3 (3)
C1—C2—C3	119.2 (3)	C15^i^—C15—C16	56.7 (3)
C1—C2—C14	120.3 (3)	C16^i^—C15—C16	26.6 (5)
C3—C2—C14	120.5 (3)	C15^i^—C15—C14	71.66 (18)
C4—C3—C2	120.0 (3)	C16^i^—C15—C14	122.4 (4)
C4—C3—C7	118.4 (3)	C16—C15—C14	108.0 (3)
C2—C3—C7	121.6 (3)	C15^i^—C15—H15B	163.3
C5—C4—C3	119.8 (3)	C16^i^—C15—H15B	80.6
C5—C4—C10	121.8 (3)	C16—C15—H15B	107.0
C3—C4—C10	118.4 (3)	C14—C15—H15B	113.9
C6—C5—C4	119.5 (3)	C15^i^—C15—H15A	83.5
C6—C5—H5	120.2	C16^i^—C15—H15A	118.9
C4—C5—H5	120.2	C16—C15—H15A	110.9
C5—C6—C1	122.7 (3)	C14—C15—H15A	108.8
C5—C6—H6	118.6	H15B—C15—H15A	108.2
C1—C6—H6	118.6	C16^i^—C16—C15^i^	96.7 (3)
O1—C7—C3	124.7 (3)	C16^i^—C16—C15	56.7 (3)
O1—C7—C8	115.5 (3)	C15^i^—C16—C15	40.0 (4)
C3—C7—C8	119.8 (3)	C16^i^—C16—C17	77.4 (2)
O2—C8—C9	124.8 (3)	C15^i^—C16—C17	125.7 (4)
O2—C8—C7	118.7 (3)	C15—C16—C17	110.5 (4)
C9—C8—C7	116.4 (3)	C16^i^—C16—H16A	164.1
C10—C9—C11	107.7 (3)	C15^i^—C16—H16A	67.7
C10—C9—C8	119.2 (3)	C15—C16—H16A	107.7
C11—C9—C8	133.1 (3)	C17—C16—H16A	108.5
O3—C10—C9	110.2 (3)	C16^i^—C16—H16B	82.2
O3—C10—C4	122.0 (3)	C15^i^—C16—H16B	122.2
C9—C10—C4	127.8 (3)	C15—C16—H16B	111.1
C12—C11—C9	104.3 (3)	C17—C16—H16B	110.5
C12—C11—C13	128.6 (3)	H16A—C16—H16B	108.5
C9—C11—C13	127.1 (3)	C18^i^—C17—C18	108.0 (3)
C11—C12—O3	112.9 (3)	C18^i^—C17—C1	109.60 (18)
C11—C12—Br1	131.9 (3)	C18—C17—C1	109.60 (18)
O3—C12—Br1	115.2 (2)	C18^i^—C17—C16^i^	98.3 (3)
C11—C13—H13A	109.5	C18—C17—C16^i^	119.9 (3)
C11—C13—H13B	109.5	C1—C17—C16^i^	110.6 (3)
H13A—C13—H13B	109.5	C18^i^—C17—C16	119.9 (3)
C11—C13—H13C	109.5	C18—C17—C16	98.3 (3)
H13A—C13—H13C	109.5	C1—C17—C16	110.6 (3)
H13B—C13—H13C	109.5	C16^i^—C17—C16	25.3 (4)
C2—C14—C15^i^	111.7 (3)	C17—C18—H18A	109.5
C2—C14—C15	111.7 (3)	C17—C18—H18B	109.5
C15^i^—C14—C15	36.7 (4)	H18A—C18—H18B	109.5
C2—C14—H14A	92.9	C17—C18—H18C	109.5
C15^i^—C14—H14A	79.0	H18A—C18—H18C	109.5
C15—C14—H14A	115.4	H18B—C18—H18C	109.5
C2—C14—H14B	119.8	C10—O3—C12	104.9 (2)
C15^i^—C14—H14B	126.1		
			
C6—C1—C2—C3	0.0	C13—C11—C12—O3	180.0
C17—C1—C2—C3	180.0	C9—C11—C12—Br1	180.0
C6—C1—C2—C14	180.0	C13—C11—C12—Br1	0.0
C17—C1—C2—C14	0.0	C1—C2—C14—C15^i^	−19.8 (2)
C1—C2—C3—C4	0.0	C3—C2—C14—C15^i^	160.2 (2)
C14—C2—C3—C4	180.0	C1—C2—C14—C15	19.8 (2)
C1—C2—C3—C7	180.0	C3—C2—C14—C15	−160.2 (2)
C14—C2—C3—C7	0.0	C2—C14—C15—C15^i^	−97.56 (14)
C2—C3—C4—C5	0.000 (1)	C2—C14—C15—C16^i^	−28.5 (5)
C7—C3—C4—C5	180.0	C15^i^—C14—C15—C16^i^	69.1 (5)
C2—C3—C4—C10	180.0	C2—C14—C15—C16	−53.3 (4)
C7—C3—C4—C10	0.0	C15^i^—C14—C15—C16	44.3 (3)
C3—C4—C5—C6	0.000 (1)	C15^i^—C15—C16—C16^i^	180.000 (2)
C10—C4—C5—C6	180.0	C14—C15—C16—C16^i^	127.6 (4)
C4—C5—C6—C1	0.0	C16^i^—C15—C16—C15^i^	180.000 (1)
C2—C1—C6—C5	0.0	C14—C15—C16—C15^i^	−52.4 (4)
C17—C1—C6—C5	180.0	C15^i^—C15—C16—C17	121.6 (4)
C4—C3—C7—O1	180.0	C16^i^—C15—C16—C17	−58.4 (4)
C2—C3—C7—O1	0.0	C14—C15—C16—C17	69.2 (4)
C4—C3—C7—C8	0.0	C6—C1—C17—C18^i^	59.21 (19)
C2—C3—C7—C8	180.0	C2—C1—C17—C18^i^	−120.79 (19)
O1—C7—C8—O2	0.0	C6—C1—C17—C18	−59.21 (19)
C3—C7—C8—O2	180.0	C2—C1—C17—C18	120.79 (19)
O1—C7—C8—C9	180.0	C6—C1—C17—C16^i^	166.5 (2)
C3—C7—C8—C9	0.0	C2—C1—C17—C16^i^	−13.5 (2)
O2—C8—C9—C10	180.0	C6—C1—C17—C16	−166.5 (2)
C7—C8—C9—C10	0.0	C2—C1—C17—C16	13.5 (2)
O2—C8—C9—C11	0.0	C16^i^—C16—C17—C18^i^	34.1 (2)
C7—C8—C9—C11	180.0	C15^i^—C16—C17—C18^i^	123.4 (5)
C11—C9—C10—O3	0.0	C15—C16—C17—C18^i^	81.0 (4)
C8—C9—C10—O3	180.0	C16^i^—C16—C17—C18	150.55 (19)
C11—C9—C10—C4	180.0	C15^i^—C16—C17—C18	−120.2 (6)
C8—C9—C10—C4	0.0	C15—C16—C17—C18	−162.6 (3)
C5—C4—C10—O3	0.0	C16^i^—C16—C17—C1	−94.83 (11)
C3—C4—C10—O3	180.0	C15^i^—C16—C17—C1	−5.6 (6)
C5—C4—C10—C9	180.0	C15—C16—C17—C1	−48.0 (4)
C3—C4—C10—C9	0.0	C15^i^—C16—C17—C16^i^	89.2 (6)
C10—C9—C11—C12	0.0	C15—C16—C17—C16^i^	46.8 (4)
C8—C9—C11—C12	180.0	C9—C10—O3—C12	0.0
C10—C9—C11—C13	180.0	C4—C10—O3—C12	180.0
C8—C9—C11—C13	0.0	C11—C12—O3—C10	0.0
C9—C11—C12—O3	0.0	Br1—C12—O3—C10	180.0

Symmetry code: (i) *x*, −*y*+3/2, *z*.

### Hydrogen-bond geometry (Å, º)

**Table:**

*D*—H···*A*	*D*—H	H···*A*	*D*···*A*	*D*—H···*A*
C6—H6···O2^ii^	0.93	2.39	3.322 (4)	177

Symmetry code: (ii) *x*+1, *y*, *z*.
